# On the Chemistry, Toxicology and Genetics of the Cyanobacterial Toxins, Microcystin, Nodularin, Saxitoxin and Cylindrospermopsin

**DOI:** 10.3390/md8051650

**Published:** 2010-05-10

**Authors:** Leanne Pearson, Troco Mihali, Michelle Moffitt, Ralf Kellmann, Brett Neilan

**Affiliations:** 1 School of Biotechnology and Biomolecular Sciences, The University of New South Wales, Sydney, NSW, 2052, Australia; E-Mails: leanne.pearson@unsw.edu.au (L.P.); troco@unsw.edu.au (T.M.); 2 School of Biomedical and Health Sciences, The University of Western Sydney, Campbelltown, NSW, 2560, Australia; E-Mail: M.Moffitt@uws.edu.au (M.M.); 3 Department of Molecular Biology, The University of Bergen, P.O. Box 7803, 5020 Bergen, Norway; E-Mail: Ralf.Kellmann@mbi.uib.no (R.K.)

**Keywords:** cyanotoxin, non-ribosomal peptide, polyketide, alkaloid, toxicology

## Abstract

The cyanobacteria or “blue-green algae”, as they are commonly termed, comprise a diverse group of oxygenic photosynthetic bacteria that inhabit a wide range of aquatic and terrestrial environments, and display incredible morphological diversity. Many aquatic, bloom-forming species of cyanobacteria are capable of producing biologically active secondary metabolites, which are highly toxic to humans and other animals. From a toxicological viewpoint, the cyanotoxins span four major classes: the neurotoxins, hepatotoxins, cytotoxins, and dermatoxins (irritant toxins). However, structurally they are quite diverse. Over the past decade, the biosynthesis pathways of the four major cyanotoxins: microcystin, nodularin, saxitoxin and cylindrospermopsin, have been genetically and biochemically elucidated. This review provides an overview of these biosynthesis pathways and additionally summarizes the chemistry and toxicology of these remarkable secondary metabolites.

## 1. Microcystin

### 1.1. Introduction

The heptapeptide hepatotoxin, microcystin, has been isolated from multiple genera of cyanobacteria, including *Microcystis, Anabaena, Oscillatoria, Planktothrix, Chroococcus* and *Nostoc*. Microcystin-producing strains such as *Microcystis aeruginosa* have a cosmopolitan distribution and thrive in a range of climates, making these organisms a global threat to human health. Consequently, significant research efforts have been directed towards their identification and eradication.

### 1.2. Chemistry

**Figure 1:**
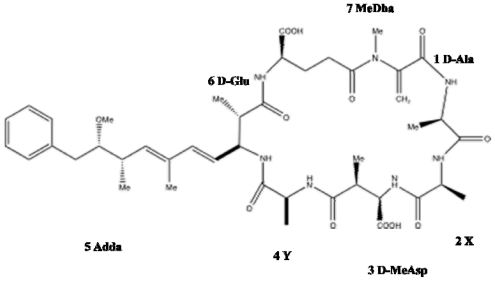
Structure of microcystin. General numbering of residues is indicated. Microcystin is a cyclic heptapeptide. The two variable amino acids in microcystin are indicated by X and Y. The most common isoform is microcystin-LR (MW 995.17), where X is l-Leu and Y is l-Arg.

The microcystins comprise the largest and most structurally diverse group of cyanobacterial toxins. Around 90 microcystin isoforms varying by degree of methylation, hydroxylation, epimerization, peptide sequence and toxicity have been identified [1,2]. Underlying the extraordinary heterogeneity present among the microcystins is their common cyclic structure (Figure 1) and possession of several rare, highly conserved amino acid moieties. Collectively, the microcystins may be described as monocyclic heptapeptides containing both d-and l-amino acids plus *N*-methyldehydroalanine and a unique ß-amino acid side-group, 3-amino-9-methoxy-2-6,8-trymethyl-10-phenyldeca-4,6-dienoic acid (Adda) [3]. The microcystin isoforms differ primarily at the two l-amino acids, and secondarily on the presence or absence of the methyl groups on d-*erythro-β*-methylaspartic acid (d-MeAsp) and/or *N-*methyldehydroalanine (Mdha) [4]. However, substitutions of all moieties within microcystin have been reported [5–7].

### 1.3. Toxicology

Acute cases of microcystin poisoning may cause rapid death in humans and other animals [8]. Upon ingestion, microcystin is transported to the liver by organic anion transport proteins where they exert their toxicity via inhibition of protein phosphatases 1 and 2A [9–11]. Inhibition of protein phosphatases can lead to excessive phosphorylation of structural filaments, subsequent cyto-skeletal degradation and breakdown of hepatic ultra structure [12,13]. Retraction of hepatocytes from neighboring cells and sinusoidal capillaries causes blood to become pooled in the liver tissues. This ultimately results in local tissue damage, organ failure and haemorrhagic shock [13].

Varying levels of toxicity have been reported for each microcystin isoform. For example, the LD50 of the most common isoform, microcystin-LR, is 50 μg per kilogram of body weight in mice [14], while the rarer microcystin-RR requires a significantly higher dose of 600 μg to produce the same lethal effect [15].

The revelation that cyanobacterial hepatotoxins cause protein phosphatase inhibition has raised the disturbing possibility that human exposure to non-lethal doses of these compounds may contribute to the development of cancer [16,17]. Several laboratory studies have indicated that chronic exposure to microcystin can indeed promote skin and liver tumors in rats and mice [18,19]. Epidemiological data suggest that similar long-term effects such as hepatocellular carcinoma may also be observed in humans [20,21]. Such results highlight the need for sensitive and rapid detection methods and stringent monitoring of cyanobacterial hepatotoxins in drinking water supplies.

### 1.4. Biosynthesis and Genetics

**Figure 2:**
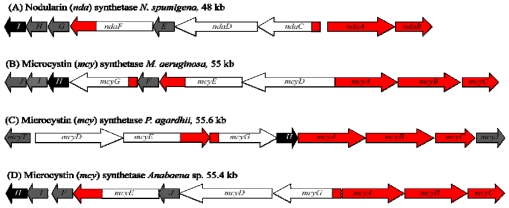
Hepatotoxin gene clusters from various cyanobacteria. Structures of the microcystin and nodularin gene clusters of (A) *N. spumigena*, (B) *M. aeruginosa,* (C) *P. agardhii,* and (D) *Anabaena* sp. 90, showing genes encoding polyketide synthases (white), non-ribosomal peptide synthetases (red), tailoring enzymes (grey), and ABC-transporters (black). Diagram not drawn to scale.

Microcystin is synthesized non-ribosomally by the thiotemplate function of a large multifunctional enzyme complex containing both non-ribosomal peptide synthetase (NRPS) and polyketide synthase (PKS) domains. The gene cluster encoding these biosynthetic enzymes, *mcyS,* has been sequenced and partially characterized in several cyanobacterial species including *Microcystis*, *Anabaena*, and *Planktothrix* [22–24] (Figure 2). Such fundamental studies have offered insight into the evolution of cyanotoxin biosynthesis, and have additionally provided much of the groundwork for current PCR-based cyanobacterial detection methods.

The microcystin biosynthesis gene cluster, *mcyS,* was the first complex metabolite gene cluster to be fully sequenced from a cyanobacterium. In *M. aeruginosa* PCC7806, the *mcyS* gene cluster spans 55 kb and comprises 10 genes arranged in two divergently transcribed operons, *mcyA–C* and *mcyD–J*. The larger of the two operons, *mcyD–J*, encodes a modular PKS (McyD), two hybrid enzymes comprising NRPS and PKS modules (McyE and McyG), and enzymes putatively involved in the tailoring (McyJ, F, and I) and transport (McyH) of the toxin. The smaller operon, *mcyA–C* encodes three NRPSs (McyA*–*C) [22].

**Figure 3:**
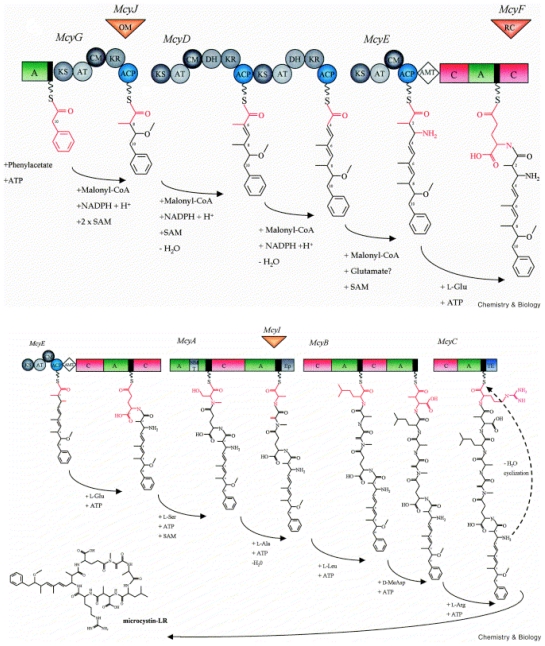
(**uper**) Model for the formation of Adda during microcystin biosynthesis and predicted domain structure of McyG, McyD and McyE. (**lower**) Biosynthetic model for microcystin-LR and predicted domain structure of McyE, McyA, McyB, and McyC. Each circle and rectangle represents, respectively, a PKS or NRPS enzymatic domain. The aminotransferase domain is represented by a diamond. The activity of the tailoring ORFs, McyJ, F and I, are shown as inverted triangles. Abbreviations are as follows: A, aminoacyl adenylation; ACP, acyl carrier protein; AMT, aminotransferase; AT, acyltransferase; C, condensation; CM, *C*-methyltransferase; DH, dehydratase; Ep, epimerization; KR, ketoacyl reductase; KS, β-ketoacyl synthase; NM, *N*-methyltransferase; OM, *O*-methyltransferase; RC, racemase; TE, thioesterase. The NRPS thiolation motif is shown in black (reproduced from [22]).

The formation of Adda putatively involves enzymes encoded by *mcyD–G* and *J*, based on bioinformatic analyses and homology to related enzymes (Figure 3a). The hybrid NRPS/PKS enzyme, McyG, constitutes the first step in Adda biosynthesis. It was initially hypothesized that the NRPS module of McyG activates phenylacetate, however, recent biochemical characterization of the McyG A–PCP didomain has revealed that assorted phenylpropanoids are preferentially activated and loaded onto the PCP [25]. Following activation, the phenylpropanoid starter unit is extended by several malonyl-CoA elongation steps and subsequently modified by *C*-methylation, reduction and dehydration, all catalyzed by the PKS modules of McyD, E and G. The aminotransferase domain of McyE then converts the polyketide to a β-amino acid in the final step of Adda biosynthesis. The NRPS module of the second hybrid PKS/NRPS enzyme, McyE, is thought to be involved in the activation and condensation of d-Glu with Adda.

The *mcyF* ORF was originally predicted to encode a glutamate racemase, responsible for the epimerization of the l-Glu residue of microcystin [22,26]. A subsequent study by [27] contended this theory and offers evidence that McyF acts exclusively as an Asp racemase. The authors propose that the d-Glu residue is provided by an l-Glu racemase residing outside the *mcyS* gene cluster. Mutagenesis experiments in *P. agardhii* showed that the production of Adda also involves an *O*-methylation step catalyzed by the putative monofunctional tailoring enzyme, McyJ [23].

The remaining biosynthetic enzymes in the microcystin biosynthesis pathway (NRPSs) are putatively involved in the specific activation, modification and condensation of substrate amino acids onto the linear peptide chain, which is then cyclized to produce microcystin. Firstly, McyA adds l-Ser to the growing chain, followed by the addition of d-Ala. This step is followed by the addition of l-Leu and d-MeAsp residues (McyB) followed by the addition of l-Arg (McyC), and subsequent cyclization and release of the final peptide product (Figure 3b).

The remaining stand alone enzyme, the 2-hydroxy-acid dehydrogenase, McyI, is putatively involved in the production of d-methylaspartate at position three within the microcystin cyclic structure via the conversion of 3-methylmalate to 3-methyloxalacetate. It is hypothesized that a promiscuous aspartate aminotransferase then converts 3-methyloxalacetate to methylaspartate [28].

An ABC transporter gene, *mcyH*, is believed to be involved in the transport of microcystin [29]. This transporter may be responsible for the thylakoid localization of the toxin [30,31] or for the extrusion of the toxin under certain growth conditions, including exposure to high and red light [32].

Comparative studies of the *mcyS* gene clusters from *M. aeruginosa, P. agardhii* [23], and *Anabaena* sp. [24] have noted variation in the arrangement of *mcyS* genes between these different species of cyanobacteria, although the proposed toxin biosynthetic processes are thought to be similar. The *M. aeruginosa* and *Anabaena* sp*. mcyS* clusters are both arranged into two divergently transcribed operons, however, the arrangement of genes within these operons differs between the two species (Figure 2). In *P. agardhii*, the *mcyS* cluster also has a distinctive arrangement and lacks *mcyF* and *mcyI.* Furthermore, the *P. agardhii mcyS* cluster contains an additional gene *mcyT,* upstream of the central promoter region. This gene is thought to encode a putative type II thioesterase enzyme, which may play an editing role by removing mis-primed amino acids from the NRPS and PKS enzymes. The characterization of *mcyS* in *M. aeruginosa*, *P. agardhii* and *Anabaena* sp. has important implications for understanding the origins and evolution of hepatotoxin biosynthesis in cyanobacteria. The identification of transposases associated with the *mcyS* and *ndaS* (nodularin) gene clusters and subsequent phylogenetic analysis has led to the theory that horizontal gene transfer and recombination events are responsible for the sporadic distribution of the *mcyS* gene cluster throughout the cyanobacteria and the various microcystin isoforms that have been identified to date [22,33,34].

Hepatotoxin production in cyanobacteria is thought to be influenced by a number of different physical and environmental parameters, including nitrogen, phosphorous, trace metals, growth temperature, light, and pH [35–41]. However, due to the fact that most regulatory investigations have not been standardized, and the data have not been interpreted against the same specific growth controls, the subject of hepatotoxin regulation remains a somewhat contentious issue. While most toxin regulation studies have focused on direct measurements of cellular toxin, the description of the *mcy* gene cluster by Tillett and co-workers [22] enabled a closer examination of microcystin regulation at the molecular level [32]. Kaebernick *et al.* used the RNase protection assay to measure the transcription of *mcyB* and *mcyD* under a variety of different light conditions. High light intensities and red light were correlated with increased transcription, while blue light led to reduced transcript levels. Interestingly, the authors observed two light thresholds, between dark and low light (0 and 16 μmol photons m^-2^ s^-1^), and medium and high light (31 and 68 μmol photons m^-2^ s^-1^), at which a significant increase in transcription occurred. The same group later found that transcription of *mcy* genes occurs via two polycistronic operons, *mcyABC* and *mcyDEFGHIJ,* from a central bidirectional promoter between *mcyA* and *mcyD* [42]. Interestingly, alternate transcriptional start sites were identified for both operons when cells were cultured under different light intensities. For example, under low light conditions, the polyketide and tailoring genes *mcyD–J* are transcribed as part of a polycistronic message (*mcyDEFGHIJ*) from a central (*mcyD*) promoter, while under high light conditions, the genes are transcribed from an alternative up-stream promoter. It is thought that initiation from the alternate promoters under high light conditions may lead to increased transcription, as previously observed for *mcyB* and *mcyD.* Many of the tailoring enzymes (*mcyF, G, H, I* and *J*) also possess their own individual promoters [32].

Interestingly, light intensity also appears to favor the production of certain microcystin variants over others. For example, Tonk *et al.* (2005) found that the cellular content of total microcystin remained constant, independent of the irradiance. However, of the two main microcystin variants detected in *P. agardhii*, the microcystin-DeRR content decreased two-fold with increased photon irradiance, whereas the microcystin-DeLR content increased three-fold. Since microcystin-DeLR is considerably more toxic than microcystin-DeRR, this implies that *P. agardhii* becomes more toxic at high light intensities [40].

Other factors such as nutrient content and temperature have also been demonstrated to affect *mcyS* expression and toxin biosynthesis. For example, Sevilla *et al.* (2008) investigated the effect of iron on *mcyS* expression and toxin biosynthesis in *M. aeruginosa* PCC7806 [41]. Real-time PCR analysis and HPLC were used to measure transcription of *mcyD* and the synthesis of microcystin-LR, respectively. The results of this study suggested that iron starvation causes an increase in *mcyD* transcription, correlative to the increase of toxin levels [41]. Davis *et al.* (2009) investigated the effects of temperature on *Microcystis* growth and toxin genes and found that elevated temperatures yield more toxic *Microcystis* cells and/or cells with more *mcyD* copies per cell, with either scenario potentially yielding more toxic blooms [39].

## 2. Nodularin

### 2.1. Introduction

The cyclic pentapeptide nodularin is most commonly isolated from the filamentous, planktonic cyanobacterium, *Nodularia spumigena.* This species generally forms toxic blooms in brackish and estuarine environments. Blooms of toxic *N. spumigena* occur annually during summer months in the Baltic Sea [43] resulting in nodularin being one of the most abundant naturally occurring compounds in the Baltic Sea. Blooms are also particularly common within the estuaries and coastal lagoons of Australia [44,45], and have been reported worldwide, including on the German North Sea coast [46], New Zealand [47], and North America [48]. *N. spumigena* scums have also been reported in the fresh to brackish/saline lakes at the mouth of the lower River Murray, South Australia, which is a major source of both potable and irrigation water [49]. Nodularin is structurally similar to microcystin and can induce similar toxic effects. The toxin has been reported to have detrimental effects on numerous organisms within the ecosystem, including invertebrates and fish, but may have no effect on other organisms [50].

The consumption of water containing toxic *N. spumigena* blooms has led to the death of domestic and native animals by massive liver haemorrhage [46,47,51,52]. In sub-acute doses, nodularin, like microcystin, is thought to act as a liver tumor initiator and promoter [53]. *N. spumigena* blooms are also of importance to the seafood industry. Nodularin has been shown to accumulate in shellfish and other seafood. Surveys of mussels, prawns, flounder both in Australia and the Baltic Sea report that nodularin accumulated to levels of concern with the potential to cause hepatotoxicity [54–56].

### 2.2. Chemistry

**Figure 4:**
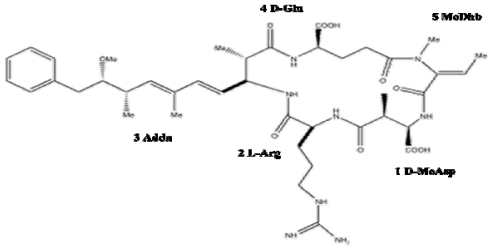
Structure of nodularin. General numbering of residues is indicated. Nodularin is a cyclic pentapeptide (MW 619). The l-Arg residue of nodularin may be replaced with a homoarginine (nodularin-Har) or valine residue (motuporin).

Nodularin is a cyclic pentapeptide with a similar structure to microcystin, consisting of Adda, d-glutamic acid (d-Glu), *N*-methyldehydrobutyrine (MeDhb), d-*erythro-β*-methylaspartic acid (d-MeAsp), and l-arginine (l-Arg) (Figure 4) [57].

Seven naturally-occurring isoforms of nodularin have been reported to date. Two of these isoforms, produced by a New Zealand *Nodularia* sp. bloom, have variations within the Adda residue, which reduces or abolishes the toxicity of the compound [5]. The d-Glu residue is essential for toxicity of nodularin, as esterification of its free carboxyl abolishes toxicity, however, substitution at position 1 has little effect on toxicity. The other two isoforms, nodularin-Har and motuporin, are variable at position 2. Nodularin-Har is produced by the strain *N. harveyana* PCC7804, with the l-Arg, replaced with l-Homoarginine (l-Har) [58,59]. Motuporin has been isolated from the Papua New Guinea sponge *Theonella swinhoei*, and may be synthesized by an associated cyanobacterium. The l-Arg residue of nodularin is replaced by l-Val in motuporin [60]. The l-Val residue is responsible for additional cytotoxicity of motuporin against cancer cell lines.

### 2.3. Toxicology

Nodularin is a potent hepatotoxin in humans and other animals. Nodularin induces liver hemorrhage in mice and has a lethal dose 50 (LD_50_) of 50 μg.kg^-1^ (intra-peritoneal route of injection) in mice [61]. At doses below this concentration, nodularin may act as a carcinogen via the initiation and promotion of liver cell division [53].

The hepatotoxicity and carcinogenicity of nodularin is associated with the inhibition of eukaryotic protein phosphatase (PP) catalytic subunit types 1 and 2A [62]. The toxin inhibits the activity of PP2A to a greater extent than PP1. Inhibition of PP2A by nodularin occurs at relatively the same concentration (IC_50_) as that of microcystin (~0.1 nM) [62]. The hydrophobic C_20_ β-amino acids Adda, present in both toxins, blocks PP enzyme activity by interacting with the hydrophobic groove and obstructing substrate access to the active site cleft [63–65]. MeDhb binds to Cys273 of PP2A in a similar fashion to the MeDha residue in microcystin which binds to Cys273 of PP1 and Cys266 of PP2A [64,66], however, binding of the toxin does not occur covalently and may be the reason for its additional carcinogenic properties [67].

The toxic effects of nodularin are primarily associated with the hepatocytes due to active transport of the toxin to the liver via the bile acid multi-specific organic anion transporters [10]. To date, studies have been unable to identify the specific mechanism of transport.

Nodularin has been observed to accumulate on different trophic levels, in numerous organisms including waterfowl, fish, mysid shrimp, zooplankton and benthic organisms [68]. Mesozooplankton in particular seem to play a major role in nodularin transfer to planktivorous fish [68]. The toxin appears to cause oxidative stress in the tissues in which it accumulates. In the case of the flounder (platichthys flesus L.), this oxidative stress occurs in the liver by way of reduced GST and CAT activities [69]. However, recent studies suggest that the toxin is rapidly detoxified and broken down or excreted [70].

### 2.4. Biosynthesis and Genetics

The nodularin biosynthesis gene cluster *ndaS*, from *Nodularia spumigena* NSOR10, was sequenced and characterized in 2004 by Moffitt and Neilan [71]. The 48 kb region of the genome consists of nine ORFs (*ndaA-I*) transcribed from a bidirectional regulatory promoter region (Figure 2). While most of the *ndaS* encoded genes have homologs in the *mcyS* cluster, their arrangement adheres more closely to the ‘co-linearity’ rule of NRPS pathways that predicts the order of catalytic processes involved in the biosynthesis of a non-ribosomal metabolite is generally the same as the order of the genes which encode their catalytic enzymes [72].

**Figure 5:**
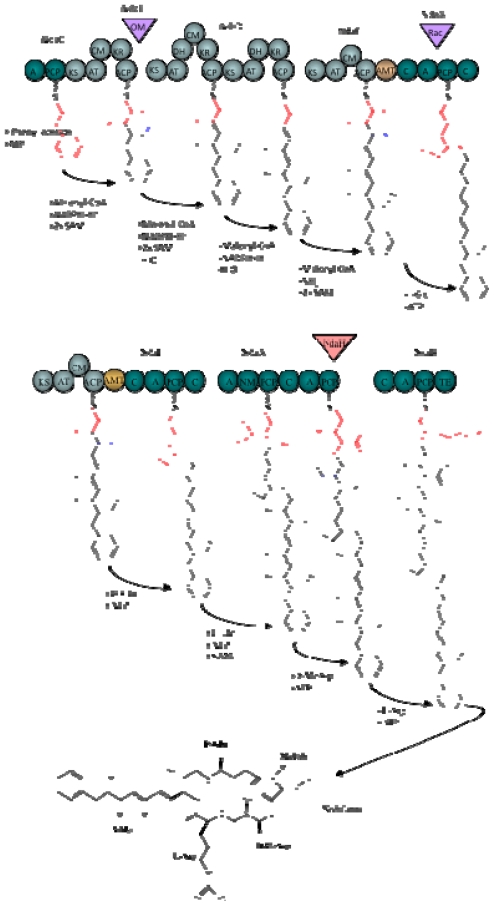
(**uper**). Model of the formation of Adda during nodularin biosynthesis and predicted domain structure of NdaC, D and F. (**lower**). Biosynthetic model for nodularin and predicted domain structure of NdaF, H, A and B. Each grey and green circle represents, respectively, a PKS or NRPS enzymatic domain. The activities of the tailoring ORFs, NdaE, G and H, are shown as inverted triangles. Abbreviations are as follows: A, aminoacyl adenylation; ACP, acyl carrier protein; AMT, aminotransferase; AT, acyltransferase; C, condensation; CM, *C*-methyltransferase; DH, dehydratase; Ep, epimerization; KR, ketoacyl reductase; KS, β-ketoacyl synthase; NM, *N*-methyltransferase; OM, *O*-methyltransferase; RC, racemase; TE, thioesterase. (reproduced from [71]).

The proposed pathway for nodularin biosynthesis is similar to that for microcystin. Functional assignment of the enzymes was based on bioinformatic analysis and homology to the microcystin synthetase enzymes. The Adda side-chain is produced via a mixed NRPS/PKS pathway from a phenylacetate starter unit and several malonyl-CoA extensions (NdaC, D and F) (Figure 5). The NRPS module of the hybrid NRPS/PKS, NdaF, subsequently adds d-Glu to the growing chain. Two NRPS enzymes, NdaA and B, complete the cyclic pentapeptide by adding the final amino acid residues, l-Thr, d-MeAsp and l-Arg. The NRPS modules responsible for the activation of d-Ala and d-Leu in *mcyS* (McyA and B) are absent from *ndaS* as nodularin lacks these moieties. The NRPS and PKS proteins require posttranslational modification by a phosphopantetheinyl transferase (PPT) protein. The PPT required for activation of the Nda proteins is not clustered with the other *nda* genes. Recently, degenerate PCR and subsequent functional enzymatic characterization, identified the PPT required for nodularin biosynthesis in *N. spumigena* NSOR10 [73].

The *ndaS* cluster also encodes several putative monofunctional tailoring enzymes that may play a role in the modification and transport of nodularin. *ndaE* encodes an *O*-methyltransferrase, *ndaG* encodes a putative l-Asp/l-Glu racemase, and *ndaI* encodes an ABC transporter. Also encoded within the *ndaS* cluster is a d-3-PGDH homolog, NdaH, which shares 71% identity with McyI. It is likely therefore, that NdaH may be involved in the production of d-MeAsp [28].

Like *mcyS*, the *ndaS* gene cluster is transcriptionally regulated by a bi-directional promoter region. Analysis of transcription of the *ndaS* cluster found that it is transcribed as two polycistronic mRNA, *ndaAB, ORF1, and ORF2,* and *ndaC* [71]. The two genes downstream of *ndaAB*, *ORF1* and *ORF2*, encode a putative transposase and a putative high light-inducible chlorophyll-binding protein, respectively. It is not clear why the putative transposase and the putative high light-inducible chlorophyll-binding protein are also co-transcribed with the *ndaS* gene cluster. *ORF2* has been identified in all strains of toxic *Nodularia* and the association between *ORF2* and nodularin biosynthesis may suggest a physiological function associated with high-light stress in the cells producing it. A putative heat shock repressor protein, encoded by the gene *ORF3*, was also identified downstream of *ORF2*, which may be involved in the transcriptional regulation of the *ndaS* genes in response to heat stress.

More recently, elucidation of the *nda* cluster has provided an opportunity to monitor transcriptional regulation of the biosynthetic pathway [74]. The effects of ammonia and phosphate starvation were analyzed. While expression of the *nda* cluster appears to be constitutive, phosphate starvation resulted in an approximately two-fold increase in expression, while ammonia supplementation decreased expression two-fold. Despite the changes to expression, intracellular and extracellular nodularin concentration remained stable [74].

## 3. Saxitoxin

### 3.1. Introduction

Saxitoxin and its analogs, collectively termed paralytic shellfish poisons (PSPs), are highly potent neurotoxins. Several freshwater species of cyanobacteria and marine dinoflagellates are known to produce saxitoxins. Blooms of these toxic species have led to mass kills of fish, native animals and livestock, as well as the contamination of freshwater resources [75–77]. Saxitoxins typically accumulate through the food chain, in organisms consumed as seafood [78]. Marine shellfish are particularly resistant to the toxins, and can therefore accumulate dangerously high levels of PSPs by ingesting toxic plankton [79]. Saxitoxin and its analogs cause an annual estimated 2000 cases of PSP globally, with a mortality rate of 15% [80].

The capacity to synthesize saxitoxins and other PSPs has an unusually wide phylogenetic distribution, including both marine and freshwater organisms from two kingdoms of life (Eubacteria and Protista). Typically, there is little convergence between the structures of secondary metabolites from marine and freshwater organisms [81], or from organisms belonging to different phylogenetic groups. Although several studies suggest that other bacteria are capable of synthesizing PSPs [82,83], the findings are controversial, as the analytical methods used were not definitive and could not be readily repeated by other researchers [84]. On the other hand, PSP biosynthesis in cyanobacteria and dinoflagellates has been extensively verified and shown to be a consistent and heritable genetic trait [85–90].

In the marine environment, dinoflagellate species capable of producing saxitoxins belong to the genera *Alexandrium*, *Pyrodinium* and *Gymnodinium* [91–93]. While in freshwater systems, several filamentous species of cyanobacteria, such as *Anabaena circinalis*, *Aphanizomenon sp.*, *Aphanizomenon gracile, Cylindrospermopsis raciborskii* and *Lyngbya wollei* are also known to produce saxitoxins [94–97]. Saxitoxin production is varied among dinoflagellate and cyanobacterial producer genera, with not all species in a toxigenic genera being toxic, and different isolates of the same species having differential toxicity. Furthermore, cyanobacterial isolates of the same species from geographically distant locations provided different toxin profiles [89].

### 3.2. Chemistry

**Figure 6:**
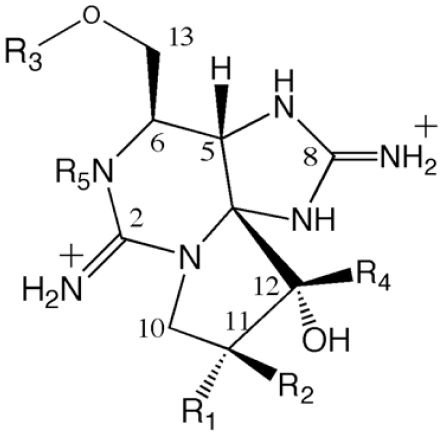
The core chemical structure of the paralytic shellfish poison (PSP), saxitoxin. ‘R’ represents variable positions. For a detailed list of isoforms see [98].

Saxitoxin is a trialkyl tetrahydropurine and the parent compound of more than 30 naturally occurring derivatives that differ structurally at four positions [98] (Figure 6.). The variable positions may be hydroxylated, sulfated or carbamoylated. Most of these analogs have been detected in both cyanobacteria and dinoflagellates [95,96,98–101]. In addition to the usual carbamate and decarbamoyl toxins, six new saxitoxin derivatives have been isolated from the cyanobacteria *Lyngbya wollei*, including derivatives which provided an acetyl side-chain instead of the typical carbamate, or lacked one of the C-12 hydroxyl moieties [102]. Further unusual modifications, such as a C-11 ethanoic acid side-chain [103], or the *N*-hydroxylation of the carbamoyl side-chain [104], have also been described in xanthid crabs. In addition, the freshwater puffer fish was shown to produce a unique saxitoxin analog where the carbamoyl side-chain is *N*-methylated [105]. Furthermore, the Panamanian golden frog *Atelopus zeteki* produces zetekitoxin AB, which was recently confirmed to be a novel analog of saxitoxin, with sodium channel blocking activity 580-fold higher than that of saxitoxin [106]. Whether or not these complex eukaryotic organisms actually produce PSPs or simply accumulate the toxins in their tissues and organs is an issue of contention.

### 3.3. Toxicology

Saxitoxin and its derivatives are the causative agents of the common seafood poisoning, paralytic shellfish poisoning (PSP). PSP symptoms generally onset within 30 minutes of ingestion, and invariably begin with a tingling or burning of the lips, tongue and throat, increasing to total numbness of the face [98]. Further symptoms may include perspiration, vomiting and diarrhea. In cases of acute poisoning, numbness may spread to the neck and extremities and progress to muscular weakness, loss of motor coordination, and finally paralysis. A lethal dose of saxitoxin usually results in cardiovascular failure due to respiratory muscle paralysis [98]. There is no clinically approved antidote to saxitoxin poisoning, and treatments during early stages of PSP include removal of unabsorbed toxin with activated charcoal, and artificial respiration. The half-life of saxitoxin in the body is approximately 90 minutes, and survival chances increase significantly after 12 hours from initial exposure [107]. It is a highly potent blocker of voltage gated sodium channels present in neuronal cell membranes [108]. In addition, saxitoxin has also been shown to block calcium channels [109], and to prolong the gating of potassium channels in heart muscle cells [110]. Saxitoxin is a highly potent phycotoxin with an intraperitoneal LD_50_ of 10 μg/kg body weight in mice [111], while human death has occurred following the ingestion of as little as 1 mg of the toxin [112].

Mammals usually contain multiple isoforms of sodium channels, with different sensitivity to saxitoxin. This difference in sensitivity is due to variation in the amino acid sequence of the channel alpha subunit, whereby a single mutation may confer insensitivity to saxitoxin, though usually sacrificing speed of gating in the process [113]. The guanidinium groups and the carbon 12 hydroxyls in STX have been shown to be critical for the binding of the sodium channel, while the carbamoyl side chain also appears to be involved in the binding process, whereby one saxitoxin molecule binds one channel by lodging itself in the ion-conducting pore [113–116].

The toxicity of saxitoxin derivatives varies greatly, with the carbamate toxins being 10–100-times more potent than the *N*-sulfo-carbamoyl derivatives [98,117]. *N*-sulfo-carbamoyl analogs are, however, labile and may easily be converted to the more toxic carbamate derivatives [118,119].

### 3.4. Biosynthesis and Genetics

**Figure 7:**
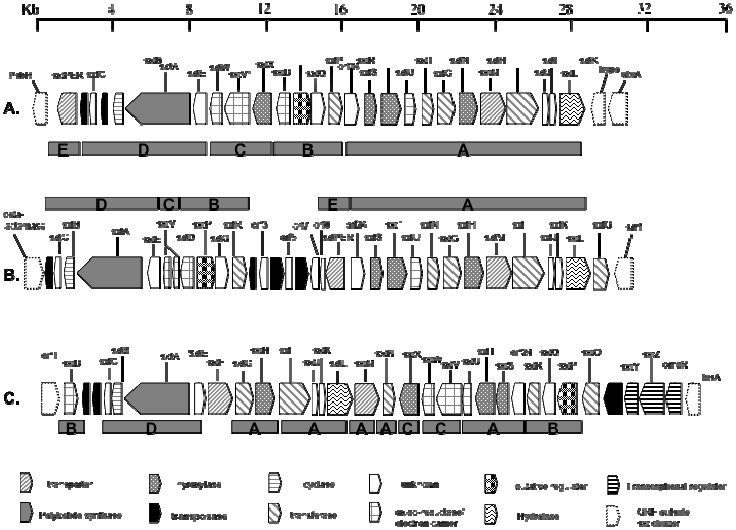
Structure of the paralytic shellfish toxin biosynthesis cluster (*sxt*) from; (a) *Aphanizomenon* sp. NH-5, (b) *Anabaena circinalis* AWQC131C, (c) *Cylindrospermopsis raciborskii* T3. Scale indicates gene length in kilobase pairs. Full bars and the letters A–E indicate common features between the various *sxt* gene clusters.

**Figure 8:**
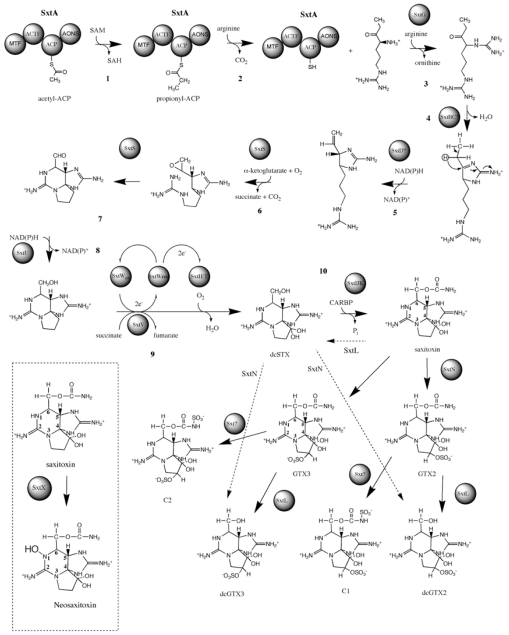
Proposed saxitoxin biosynthetic pathway in cyanobacteria based on intermediate characterization and bioinformatic analysis. Dashed lines indicate possible alternative reactions [see text for detailed steps].

Using a reverse genetic approach, Kellmann and co-workers [120] identified the gene cluster putatively responsible for the biosynthesis of saxitoxin in *Cylindrospermopsis raciborskii* T3 (*sxt*) (Figure 7c). The *sxt* gene cluster is encoded by more than 35 kb and comparative sequence analysis assigns 30 catalytic functions to 26 proteins. Bioinformatic analysis of this cyanobacterial saxitoxin gene cluster, coupled with identification of novel biosynthetic intermediates enabled a revision of the previously proposed saxitoxin biosynthesis pathway (Figure 8).

The first step in the revised saxitoxin biosynthesis pathway involves a Claisen condensation reaction catalyzed by SxtA. This unique enzyme possesses a polyketide synthase (PKS)-like structure composed of four catalytic domains, SxtA1-4: SxtA1 is homologous to SAM-dependant methyltransferases; SxtA2 is related to GCN5-related *N*-acetyl transferases (GNAT) that transfer acetate from acetyl-CoA to various heteroatoms [121]; SxtA3 is related to ACPs and also provides a phosphopantetheinyl-attachment site; SxtA4 is homologous to class II aminotransferases and is most similar to AONS (8-amino-7-oxononanoate synthase). The predicted reaction sequence of SxtA, based on its primary structure, is the loading of the ACP (SxtA3) with acetate from acetyl-CoA, followed by the SxtA1-catalyzed methylation of acetyl-ACP, converting it to propionyl-ACP. SxtA4, the class II aminotransferase domain, then performs a Claisen condensation reaction between propionyl-ACP and arginine. The putative product of SxtA is thus 4-amino-3-oxo-guanidinoheptane, which Kellmann *et al.* designated compound A.

The *sxtG* gene encodes a putative amidinotransferase, with highest amino acid sequence similarity to l-arginine/l-lysine amidinotransferases. SxtA is the putative substrate for SxtG, which transfers an amidino group from arginine to the α-amino A′ group, thus producing 4,7-diguanidino-3-oxoheptane (designated compound B′). SxtB, an enzyme similar to the cytidine deaminase-like enzymes from gammaproteobacteria, then catalyzes a retroaldol-like condensation in the conversion from B′ to C′.

The putative sterol desaturase, SxtD is predicted to introduce a double bond between C-1 and C-5 of C′, resulting in the 1,2-H shift between C-5 and C-6 (compound D′). The gene product of *sxtS,* which has sequence homology to nonheme iron 2-oxoglutarate-dependent dioxygenases, is predicted to perform the consecutive epoxidation of the new double bond and opening of the epoxide to an aldehyde with concomitant bicyclization. SxtU has sequence similarity to short-chain alcohol dehydrogenases and is therefore predicted to reduce the terminal aldehyde group of the saxitoxin precursor forming compound E′. The concerted action of SxtD, SxtS, and SxtU is therefore responsible for the hydroxylation and bicyclization of compound C′ to E′.

The gene product of *sxtI* is most similar to a predicted *O*-carbamoyltransferase from *Trichodesmium erythraeum* and other cyanobacteria. Kellmann *et al.*’s data [120] indicate that SxtI may catalyze the transfer of a carbamoyl group from carbamoylphosphate to the free hydroxy group of E′. Adjacent to *sxtI* are two short ORFs of unknown function*, sxtJ* and *sxtK.* While *sxtJ and sxtK* homologs are available in the databases, none of these genes have been functionally characterized.

*sxtH* and *sxtT*, each encode a terminal oxygenase subunit similar to those found in bacterial phenylpropionate and related ring-hydroxylating dioxygenases. SxtH and SxtT may therefore perform the consecutive hydroxylation of C-12, converting F′ into saxitoxin. Members belonging to bacterial phenylpropionate and related ring-hydroxylating dioxygenases are multicomponent enzymes, as they require an oxygenase reductase for their regeneration after each catalytic cycle. The *sxt* gene cluster provides a putative electron transport system, which would fulfill this function in the form of SxtV and SxtW. SxtV, a 4Fe-4S ferredoxin, could putatively extract an electron pair from succinate, converting it to fumarate [122]. SxtW a fumarate/reductase/succinate dehydrogenase homolog could then transfer the electrons via ferredoxin to SxtH and SxtT.

Following synthesis of the parent molecule saxitoxin, modifying enzymes introduce various functional groups. In addition to saxitoxin, *C. raciborskii* T3 produces *N-*1-hydroxylated (neoSTX), decarbamoylated (dcSTX), and *N*-sulfurylated (GTX-5) toxins, whereas *Anabaena circinalis* AWQC131C produces decarbamoylated (dcSTX) toxins and *O*-sulfurylated (GTX-3/GTX-2, dcGTX-3/dcGTX-2) toxins, as well as both *O*-and *N*-sulfurylated toxins (C-1/C-2), but no *N*-1-hydroxylated toxins [99].

*sxtX* encodes an enzyme with homology to cephalosporin hydroxylase. *sxtX* was detected only in *C. raciborskii* T3, *Aphanizomenon flosaquae* NH-5, and *Lyngbya wollei*, which produce *N*-1-hydroxylated analogs of saxitoxin [94,108,123], such as neoSTX. This component of the gene cluster was not present in any strain of *A. circinalis*, and therefore probably represents the reason why this species does not produce *N*-1-hydroxylated PSP toxins [89,99]. The predicted function of SxtX is therefore the *N*-1 hydroxylation of saxitoxin.

*A. circinalis* AWQC131C and *C. raciborskii* T3 also produce *N*-and *O*-sulfated analogs of saxitoxin [GTX-5, C-2/C-3, (dc)GTX-3/GTX-4]. The activity of two 3′-phosphate 5′-phosphosulfate (PAPS)-dependent sulfotransferases, which were specific for the *N*-21 of saxitoxin and GTX-3/GTX-2 and the *O*-22 of 11-hydroxy saxitoxin, respectively, has been described previously in studies of the PSP toxin-producing dinoflagellate *Gymnodinium catenatum* [124,125]. A putative sulfotransferase encoded by *sxtN* is predicted to transfer a sulfate group to either *N*-21 or *O*-22. Interestingly, the *sxt* gene cluster also encodes an adenylylsulfate kinase (APSK), SxtO, putatively involved in the formation of PAPS. Other biosynthetic gene clusters that result in sulfated secondary metabolites also contain genes required for the production of PAPS [126].

Decarbamoylated analogs of STX could be produced via either of two hypothetical scenarios. Enzymes that act downstream of SxtI, the carbamoyltransferase, in the biosynthesis of PSP toxins are proposed to exhibit broad substrate specificity, processing both carbamoylated and decarbamoylated precursors of STX. Alternatively, hydrolytic cleavage of the carbamoyl moiety from STX or its precursors may occur. SxtL is related to GDSL lipases, which are multifunctional enzymes with thioesterase, arylesterase, protease, and lysophospholipase activities [127]. The function of SxtL could therefore include the hydrolytic cleavage of the carbamoyl group from STX analogs.

Kinetic studies of PSP toxin accumulation in producing cells and the media of cyanobacterial cultures suggest that there is an active transport mechanism for these toxins [85]. In addition, variations in the concentration of sodium in culture media are known to affect the accumulation of PSP toxins in producer cells [128]. *sxtF* and *sxtM* encoded two proteins with high sequence similarity to sodium-driven multidrug and toxic compound extrusion (MATE) proteins of the NorM family. Members of the NorM family of MATE proteins are bacterial sodium-driven antiporters that export cationic substances [129]. All of the PSP toxins are cationic substances, except for the C toxins, which are zwitterionic. It is therefore probable that SxtF and SxtM are also involved in the export of PSP toxins.

Environmental factors such as nutrient (e.g., nitrogen and phosphate) content, salinity and temperature have been reported to regulate the production of PSP toxins in dinoflagellates and cyanobacteria [130–132]. Two transcriptional factors, *sxtY* and *sxtZ*, related to PhoU and OmpR, respectively, as well as a two-component regulator histidine kinase proximal to the 3′ end of the *sxt* gene cluster in *C. raciborskii* T3 have been identified. PhoU-related proteins are negative regulators of phosphate uptake [133], whereas OmpR-like proteins are involved in the regulation of a variety of metabolisms, including nitrogen [134] and osmotic balance [135]. It is therefore likely that PSP toxin production in *C. raciborskii* T3 may be regulated at the transcriptional level in response to the availability of phosphate as well as other environmental factors.

Following the identification and characterisation of the *sxt* cluster from *C. raciborskii*, Mihali *et al.* [136] have described similar gene clusters from an Australian isolate of *Anabaena circinalis* and an American isolate of *Aphanizomenon sp.* (Figure 7b and a, respectively). These saxitoxin gene clusters are slightly smaller than the *C. raciborskii*, spanning approximately 28 kb. The topology of all three *sxt* clusters is also varied which suggests the occurrence of multiple transposition events throughout the evolution of saxitoxin biosynthesis in the cyanobacteria. Phylogenetic analysis of the *sxt O*-carbamoyltransferase gene across several saxitoxin producing species indicated that the most likely origin of the gene was an ancestral a-proteobacterium and that the entire set of genes required for saxitoxin biosynthesis probably spread by horizontal gene transfer [137].

## 4. Cylindrospermopsin

### 4.1. Introduction

The cyanobacterial alkaloid toxin, cylindrospermopsin, was first identified in 1979 when 148 people were hospitalized with symptoms of hepatoenteritis on Palm Island (Queensland, Australia). This outbreak was later linked to a bloom of *Cylindrospermopsis raciborskii* in a drinking water reservoir [138,139]. In addition to its impact on human health, cylindrospermopsin poisonings have been linked to the death of domestic animals [140].

Eight cyanobacterial species have thus far been identified as cylindrospermopsin producers; *Cylindrospermopsis raciborskii*, *Aphanizomenon ovalisporum*, *Aphanizomenon flosaquae*, *Umezakia natans*, *Rhaphdiopsis curvata* and *Anabaena bergii, Anabaena lapponica,* and *Lygnbya wollei* [141–148]. The wide distribution of cylindrospermopsin producing species, coupled with the invasiveness of the chief toxin producer, *C. raciborskii*, presents a major problem for water management, on a global scale [149].

### 4.2. Chemistry

**Figure 9:**
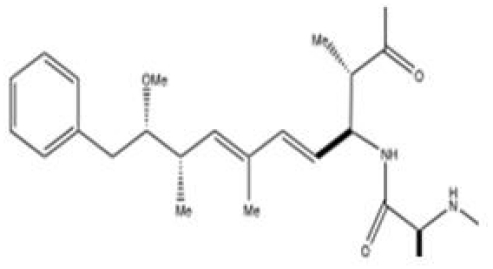
The chemical structure of the NRPS and PKS derived alkaloid cylindrospermopsin. Natural variants containing an epimer at the hydroxyl bridge (C7) or lacking the hydroxyl altogether have also been reported [151].

Cylindrospermopsin is a polyketide-derived alkaloid with a central functional guanidino moiety and a hydroxymethyluracil attached to the tricyclic carbon skeleton [150] (Figure 9). The natural occurrence of an epimer at the hydroxyl bridge, 7-epicylindrospermopsin [151], and a cylindrospermopsin variant lacking the hydroxyl group at C7, 7-deoxycylindrospermopsin, have also been reported [143].

### 4.3. Toxicology

Cylindrospermopsin is a highly biologically active alkaloid, interfering with several metabolic pathways. It has hepatotoxic, general cytotoxic [152–154] and neurotoxic [155] effects and is considered a potential carcinogen [156]. The toxicity of cylindrospermopsin is mediated through the inhibition of glutathione, protein synthesis and cytochrome P450 [152–154,157], with the uracil moiety as well as the hydroxyl at C7 being crucial for toxicity [158,151]. In mammals, cylindrospermopsin poisoning can cause liver, kidney, thymus and heart damage [159,160].

### 4.4. Biosynthesis and Genetics

**Figure 10:**
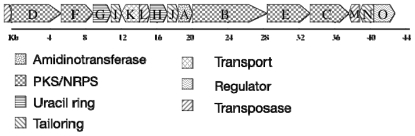
Structural organization of the cylindrospermopsin gene cluster from *C. raciborskii* AWT205. Scale indicates gene cluster in base pairs.

**Figure 11:**
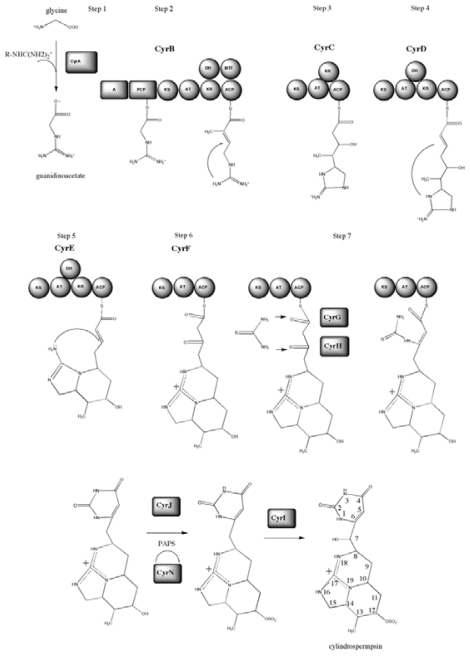
Proposed biosynthetic pathway for cylindrospermopsin (see text for detail).

The cylindrospermopsin biosynthesis (*cyr*) gene cluster from *C. raciborskii* AWT205 was recently sequenced [161] The cluster spans 43 kb and contains 15 ORFs, which encode all the functions required for the biosynthesis, regulation and export of the toxin (Figure 10). Biosynthesis is initiated via an amidinotransfer onto glycine followed by five polyketide extensions and subsequent reductions, rings are formed via Michael additions in a step-wise manner. The uracil ring is formed by a novel pyrimidine biosynthesis mechanism and tailoring reactions, including sulfation and hydroxylation that complete biosynthesis (Figure 11).

The first step in formation of the carbon skeleton of cylindrospermopsin involves the synthesis of guanidinoacetate via the transamidination of glycine [162–164]. An amidinotransferase encoded by *cyrA*, putatively transfers a guanidino group from arginine [163], to glycine thus forming guanidinoacetate. A mixed NRPS-PKS encoded by *cyrB* is thought to activate guanidinoacetate, which is then transferred via the swinging arm of the peptidyl carrier protein (PCP) to the KS domain. The AT domain of CyrB activates malonyl-CoA and attaches it to the ACP. This is followed by a condensation reaction between the activated guanidinoacetate and malonyl-CoA in the KS domain. The methyl transferase (MT) domain identified in CyrB is predicted to methylate C13. CyrB contains two reducing modules, KR and DH. Their concerted reaction reduces the keto group to a hydroxyl followed by elimination of H_2_O, resulting in a double bond between C13 and C14. A nucleophilic attack of the amidino group at N19 onto the newly formed double bond between C13 and C14 then putatively occurs via a ‘Michael addition’. The cyclization follows Baldwin’s rules for ring closure [165], resulting in the formation of the first ring in cylindrospermopsin. This reaction could be spontaneous and may not require enzymatic catalysis, as it is energetically favorable [165]. This is the first of three ring formations and is one of the principal differences between Mihali *et al.*’s [161] biosynthetic pathway and that previously proposed [163].

The third step in the biosynthesis of cylindrospermopsin involves *cyrC,* which encodes a PKS with KS, AT, KR, and ACP domains. The action of these domains results in the elongation of the growing chain by an acetate via activation of malonyl-CoA by the AT domain, its transfer to ACP and condensation at the KS domain with the product of CyrB. The elongated chain is bound to the ACP of CyrC and the KR domain reduces the keto group to a hydroxyl group on C12. Following the catalysis of enzyme CyrC is CyrD, a PKS. The action of this PKS module on the product of CyrC results in the addition of one acetate and the reduction of the keto group on C10 to a hydroxyl and dehydration to a double bond between C9 and C10. This double bond is the site of a nucleophilic attack by the amidino group N19 via another Michael addition that again follows Baldwin’s rules of ring closure [165], resulting in the formation of the second ring, the first six-membered ring made in cylindrospermopsin. The intermediate produced by CyrD is the substrate for CyrE (step 5 in Figure 1). A PKS, CyrE, catalyzes the addition of one acetate and the formation of a double bond between C7 and C8. This double bond is attacked by N18 via a Michael addition and the third cyclisation occurs, resulting in the second 6-member ring. The *cyrF* gene encodes the final PKS module -a minimal PKS containing only a KS, AT, and ACP. CyrF acts on the product of CyrE and elongates the chain by an acetate, leaving C4 and C6 unreduced. Step 7 in the pathway involves the formation of the uracil ring, a reaction that has been elusive so far and is required for the toxicity of the final cylindrospermopsin compound [151].

The cylindrospermopsin gene cluster encodes two enzymes CyrG and CyrH that are most similar to the enzyme family of amidohydrolases/ureases/dihydrotases, whose members catalyze the formation and cleavage of N-C bonds. Mihali and co-workers [161] propose that these enzymes transfer a second guanidino group from a donor molecule, such as arginine or urea, onto C6 and C4 of cylindrospermopsin resulting in the formation of the uracil ring. The first reaction consists of the formation of a covalent bond between the N of the guanidino donor and C6 of cylindrospermopsin followed by an elimination of H_2_O forming a double bond between C5 and C6. The second reaction catalyses the formation of a bond between the second N on the guanidino donor and C4 of cylindrospermopsin, co-committently with the breaking of the thioester bond between the acyl carrier protein of CyrF and cylindrospermopsin, causing the release of the molecule from the enzyme complex. The third reaction -if required -would catalyze the cleavage of the guanidino group from a donor molecule other than urea. The action of CyrG and CyrH in the formation of the uracil ring in cylindrospermopsin describes a novel biosynthesis pathway of a pyrimidine. Mihali and co-workers’ [161] genetic analysis shows that cyclization may happen stepwise, with successive ring formation of the appropriate intermediate as it is synthesized. This mechanism also explains the lack of a thioesterase or cyclization domain, which are usually associated with NRPS/PKS modules and catalyze the release and cyclization of the final product from the enzyme complex.

The sulfation of cylindrospermopsin at C12 is likely to be carried out by the action of a sulfotransferase. The *cyrJ* gene encodes a protein that is most similar to human 3′-phosphoadenylyl sulfate (PAPS) dependent sulfotransferases. Similar enzymes have recently been implicated in the sulfation of other cyanotoxins [120]. The cylindrospermopsin gene cluster also encodes an adenylsulfate kinase (ASK), namely CyrN. ASKs are enzymes that catalyse the formation of PAPS, which is the sulfate donor for sulfotransferases. Mihali and co-workers [161] propose that CyrJ sulfates cylindrospermopsin at C12 while CyrN creates the pool of PAPS required for this reaction. Screening of cylindrospermopsin producing and non-producing strains revealed that the sulfotransferase genes were only present in cylindrospermopsin producing strains, further affirming the involvement of this entire cluster in the biosynthesis of cylindrospermopsin. The *cyrJ* gene might therefore be a good candidate for a toxin probe, as it is more unique than NRPS and PKS genes and would presumably have less cross-reactivity with other gene clusters containing these genes, which are common in cyanobacteria. The final tailoring reaction is carried out by CyrI. CyrI putatively catalyzes the hydroxylation of C7, a residue that, along with the uracil ring, seems to confer much of the toxicity of cylindrospermopsin [158,166].

The cylindrospermopsin gene cluster contains an ORF denoted *cyrK*, the product of which is most similar to sodium ion driven multi-drug and toxic compound extrusion proteins (MATE) of the NorM family. CyrK is hypothesized to function as a transporter for cylindrospermopsin, based on this homology and its central location in the cluster.

Cylindrospermopsin production has been shown to be highest when fixed nitrogen is eliminated from the growth media [167]. Flanking the cylindrospermopsin gene cluster are “*hyp*” gene homologs involved in the maturation of hydrogenases. In the cyanobacterium *Nostoc* PCC73102 they are under the regulation of the global nitrogen regulator NtcA, that activates transcription of nitrogen assimilation genes [168,169]. It is plausible that the cylindrospermopsin gene cluster is under the same regulation, as it is located wholly within the “*hyp*” gene cluster in *C. raciborskii* AWT205, and no obvious promoter region in the cylindrospermopsin gene cluster could be identified. Finally, the cylindrospermopsin cluster also includes an ORF at its 3’-end designated CyrO. By homology, it encodes a hypothetical protein that appears to possess an ATP binding cassette, and is similar to WD repeat proteins, which have diverse regulatory and signal transduction roles. CyrO may also have a role in transcriptional regulation and DNA binding. It also shows homology to AAA family proteins that often perform chaperone-like functions and assist in the assembly, operation, or disassembly of protein complexes. Further insights into the role of CyrO are hindered due to low sequence homology with other proteins in databases.
